# S100B Blood Level Determination for Early Management of Ski-Related Mild Traumatic Brain Injury: A Pilot Study

**DOI:** 10.3389/fneur.2020.00856

**Published:** 2020-08-14

**Authors:** Samy Kahouadji, Pauline Salamin, Laurent Praz, Julien Coiffier, Vincent Frochaux, Julie Durif, Bruno Pereira, Lionel Arlettaz, Charlotte Oris, Vincent Sapin, Damien Bouvier

**Affiliations:** ^1^Biochemistry and Molecular Genetic Department, CHU Clermont-Ferrand, Clermont-Ferrand, France; ^2^Department of Emergency Medicine, Valais Hospital, Sion, Switzerland; ^3^Biostatistics Unit (DRCI), CHU Clermont-Ferrand, Clermont-Ferrand, France; ^4^Department of Biology, ICH, Valais Hospital, Sion, Switzerland; ^5^Université Clermont Auvergne, CNRS, INSERM, GReD, Clermont-Ferrand, France

**Keywords:** S100B, mTBI (mild traumatic brain injury), ski, ski accidents, biomarker

## Abstract

**Background:** Mild traumatic brain injury (mTBI) management in emergency departments is a complex process involving clinical evaluation, laboratory testing, and computerized tomography (CT) scanning. Protein S100B has proven to be a useful blood biomarker for early evaluation of mTBI, as it reduces the required CT scans by one-third. However, to date, the ability of S100B to identify positive abnormal findings in the CT scans of patients suffering from mTBI caused by ski practice has not been investigated. Thus, the primary aim of this study was to investigate the diagnostic performance of S100B as an mTBI management biomarker in patients with ski-related mTBI.

**Materials and Methods:** One hundred and thirty adult mTBI patients presenting to the emergency department of Hôpital du Valais in Sion, Switzerland, with a Glasgow Coma Scale (GCS) score of 13–15 and clinical indication for a CT scan were included in the study. Blood samples for S100B measurement were collected from each patient and frozen in 3-hour post-injury intervals. CT scans were performed for all patients. Later, serum S100B levels were compared to CT scan findings in order to evaluate the biomarker's performance.

**Results:** Of the 130 included cases of mTBI, 87 (70%) were related to ski practice. At the internationally established threshold of 0.1 μg/L, the receiver operating characteristic curve of S100B serum levels for prediction of abnormal CT scans showed 97% sensitivity, 11% specificity, and a 92% negative predictive value. Median S100B concentrations did not differ according to sex, age, or GCS score. Additionally, there was no significant difference between skiers and non-skiers. However, a statistically significant difference was found when comparing the median S100B concentrations of patients who suffered fractures or had polytrauma and those who did not suffer fractures.

**Conclusion:** The performance of S100B in post-mTBI brain lesion screenings seems to be affected by peripheral lesions and/or ski practice. The lack of neurospecificity of the biomarker in this context does not allow unnecessary CT scans to be reduced by one-third as expected.

## Introduction

Mild traumatic brain injury (mTBI) is a complex injury that causes a wide range of symptoms and disabilities. It constitutes a major public health concern, as it leads to a significant amount of morbidity and mortality worldwide. Although there are differences in criteria used to categorize traumatic brain injuries (1), these injuries are usually stratified using the Glasgow Coma Scale (GCS), which differentiates between severe (score of 3–8), moderate (score of 9–12), and mild brain (mTBI) injuries (score of 13–15). About 70–90% of all treated brain injuries are mTBIs, with an incidence estimated at 100–300 per 100,000 people (2, 3). The two leading causes of mTBI-related hospital admissions in Europe and the US are road traffic accidents and falls (3, 4). Different mechanisms of mTBI, associations with peripheral bone fractures and sport practice could influence patients' management in the emergency department (ED). In Allouchery et al.'s (5) study of 1,449 mTBI patients admitted to Clermont-Ferrand's adult ED, 59% were linked to a domestic fall, 14% to a road accident, and 6% to a sport-related accident. The proportions of these causes may vary depending on the location of the hospital. For example, in hospitals located near major ski resorts, winter-sport-related head trauma constitutes an important portion of emergency admissions. Indeed, in the 2018 Club Alpin Suisse (CAS) report, the Swiss mountain emergency medicine organizations Garde aérienne Suisse de sauvetage (Rega), Secours Alpin Suisse (SAS), and Organisation Cantonale Valaisanne de Secours (OCVS) registered 3,211 accidents, 729 of which were related to winter sports (6).

Computerized tomography (CT) scans of the brain are essential for diagnostic work-up, classification, prognostication, and follow-up of mTBI patients (7). Additionally, such scans enable detection of patients who require neurosurgical care. Intracranial complications occur in 10% of mTBI patients, and 1% of cases require neurosurgical intervention, with a 0.1% fatality rate (8). Screening all mTBI patients would be expensive, and the ionizing radiation involved in screening incurs potential risks (9). Limiting unnecessary CT scans is essential to reduce overcrowding in EDs, avoid exposing patients to radiation, and achieve significant financial savings (10). The decision to perform imaging in cases of head trauma depends on multiple factors, including clinical symptoms and measured blood biomarkers (11). Although clinical parameters, including headache, nausea, vomiting, amnesia, and seizures, may be correlated with positive CT scans, the number of negative CTs is not significantly reduced when they are present (12–14).

S100 calcium-binding protein B (S100B) is the most commonly studied blood biomarker for its ability to predict abnormal findings in the CT scans of mTBIs in children and adults (15, 16). Since 2013, S100B has been implemented in Scandinavian countries for management of mTBI (17). Upon admission, measurement of S100B serum may reduce 30% of unnecessary negative CT scans (16, 18–20). In addition, there is a growing interest of S100B amongst other head trauma biomarkers in the management of sport-related concussions (21), as consequences of repeated mTBIs have been linked to brain lesions similar to those observed in dementia (22–24).

As of today, S100B is the only blood biomarker used in routine mTBI screening guidelines in Europe and the ~30% CT scans reduction has been established in routine use (5, 17, 20). However, we do not observe the same performances in our practice in the ED of Valais Hospital in Sion, Switzerland, where a relatively high percentage of brain injury admissions is ski-related. S100B has never been studied in the context of ski-related mTBI, and there is no literature that provides data on the influence of ski practice on serum S100B concentrations. We conducted this study to evaluate the diagnostic performances of serum S100B measurement to identify CT scan abnormalities (i.e., positive CT scans) in a cohort of mTBI patients in which ski-related accidents represent a significant percentage. The main outcome of this study is to assess the CT scan reduction allowed by S100B in this population of patients.

## Materials and Methods

### Study Design

This prospective study was carried out from February 2018 to April 2019 at the Department of Emergency Medicine at Valais Hospital (*Centre Hospitalier du Valais Romand*) in Sion, Switzerland. The study was approved by CER-VD and conducted in accordance with the ethical principles for medical research outlined in the Helsinki Declaration. Patients provided an informed consent. Adult (18 years old and older) mTBI patients with a clinical indication for a CT scan, as described in the Canadian CT Head Rule (25) were included. mTBI patients were defined as patients suffering from a head trauma with a GCS score of 13–15, as determined by the attending physician. All patients underwent a CT scan and venipuncture for subsequent S100B and creatine kinase (CK) blood measurements. The time interval for S100B sampling was set at 3 h post-trauma, as described by Biberthaler et al. (18) and Laribi et al. (26).

### Data Collected

Data collected were: sex, age, GCS score, mechanism of injury (occurred during alpine skiing, other sport-related accident, road accident, domestic accident, alcohol intoxication and other), the wear of a safety helmet, ski session duration, presence of fractures and if present, the number of fractures, and whether the patients were described as polytraumatized according to Lew et al. (27).

### S100B and Creatine Kinase Assays

Venous blood samples were centrifuged at 2,100 g for 15 min and stored at −20°C until analysis. The S100B results did not influence patients' clinical management.

Serum S100B concentrations were determined by an electro-chemiluminescence immunoassay using a Roche Diagnostics Cobas e411® instrument (Meylan, France). The assay time was 18 min, the sample volume was 20 μL, and the lower detection limit was 0.005 μg/L. Concentrations of up to 39 μg/L could be measured without dilution. Typical within-assay precision was below 5%. The results are reported in micrograms per liter and rounded to two decimal places. Total CK activity concentration assays were performed using a Vista® analyzer (Siemens, Munich, Germany) following the manufacturer's recommendations.

### Cranial Computed Tomography Scans

An emergency cranial CT scan was performed using a GE Healthcare Revolution GSI® according to the following protocol: helical mode with a slice thickness of 2.25 mm, an interval of 1.25 mm, 120 kV, and a maximum of 280 mA from C1 to the top of the head with additional bone window reconstructions. All CT scans were analyzed by a radiologist. To determine whether patients had a trauma-relevant intracranial lesion, radiological parameters were recorded. The patients were divided into two groups: normal CT scan (CT–) for mTBI patients with no signs of trauma-relevant intracranial lesions and abnormal CT scan (CT+) for mTBI patients with at least one pathophysiological trauma-relevant intracranial lesion. CT scans were considered positive if any signs of cranial (skull fracture) or intracranial pathology (hematoma, air, or contusion) were present, subgaleal hematomas were also considered positive to prevent disregarding abnormalities that may influence S100B levels.

### Statistics

Statistical analyses were performed using Stata software, version 15 (StataCorp, College Station, US). The tests were two-sided, with the Type I error set at 5%. Continuous data were expressed as the mean ± standard deviation (SD) or median (interquartile range) according to the statistical distribution. The assumption of normality was assessed using the Shapiro-Wilk test. An analysis of variance (ANOVA) or Kruskal-Wallis test (when ANOVA assumptions were not met) were performed to compare continuous parameters (e.g., S100B serum) between the independent groups. The assumption of homoscedasticity was verified using Bartlett's test. Then, to evaluate the ability of S100B serum to identify CT+ patients, the receiver operating characteristic (ROC) curve was plotted. The area under the curve was estimated with a 95% confidence interval.

Univariate analyses were completed using several inferential statistical tests. Comparisons involving categorical variables were performed with the chi-squared or Fisher's exact tests, while the relationships between continuous variables were explored using either Pearson's or Spearman's correlation coefficient, depending on the statistical distribution. The correlation results were illustrated with a color-coded heatmap.

Multivariate analyses, specifically multiple linear regressions, were carried out to investigate the influence of different variables on S100B levels. The normality of residuals was examined as mentioned above, and logarithmic transformation of S100B serum concentrations was proposed to achieve the normality assumption. Sensitivity analyses were performed to guarantee the robustness of the results.

## Results

### Patient Characteristics

Between February 2018 and April 2019, a total of 130 patients admitted for head trauma in the ED were recruited for this study. S100B serum assays were performed within 3 h of the injury. The sample included 81 males (62%) and 49 females (38%), with a 1.6 sex ratio (male/female). The mean age was 44.8 years (SD: 20.4). In total, 108 (83%) patients had a GCS score of 15 at admission, and 22 (17%) had a GCS score of 13 or 14. Of the 130 patients, 90 (69%) had a sport-related accident, 87 of them were ski-related. The helmet was used by 76 of the 87. The mean ski session duration was 2 h and 18 min (SD: 1 h and 42 min). Thirty-three (25%) patients had abnormal findings in the initial CT scan (CT+; Table 1). The pathophysiological trauma-relevant findings were as follows: subgaleal hematoma (45%), subarachnoid hemorrhage (24%), basal skull fracture (18%), intraparenchymal hemorrhage (9%), and intracranial hemorrhage (3%). The median serum S100B level was 0.21 μg/L (min: 0.05; max: 1.42; IQR: 0.14–0.35).

**Table 1:** Demographic characteristics, clinically relevant information, and radiological findings for the whole study population.

	**Data**
Total	130
Sex ratio (M/F)	1.6
Mean age in years (SD)	44.8 (20.4)
**GCS at admission**	
15	108 (83%)
13–14	22 (17%)
Positive CT scan	33 (25%)
Subgaleal hematoma	15 (45%)
Subarachnoid hemorrhage	8 (24%)
Basal skull fracture	6 (18%)
Intraparenchymal hemorrhage	3 (9%)
Intracranial hemorrhage	1 (3%)
Contusions at admission	59 (45%)
Fractures at admission	34 (26%)
Fracture = 1	26 (20%)
Fractures ≥ 2	8 (6%)
Polytrauma	12 (9%)
**Injury mechanism**	
Road accident	13 (10%)
Ski-related accident	87 (67%)
Other sport-related accident	3 (2%)
Domestic accident	14 (11%)
Alcohol intoxication	5 (4%)
Other	6 (5%)

*GCS, Glasgow Coma Scale; CT scan, computed tomography scan; fractures = 1, patients that suffered only one fracture; fractures ≥ 2, patients that suffered at least two fractures; SD, standard deviation*.

### S100B Performance for Identifying Abnormal Findings in CT Scans

**Figure 1:**
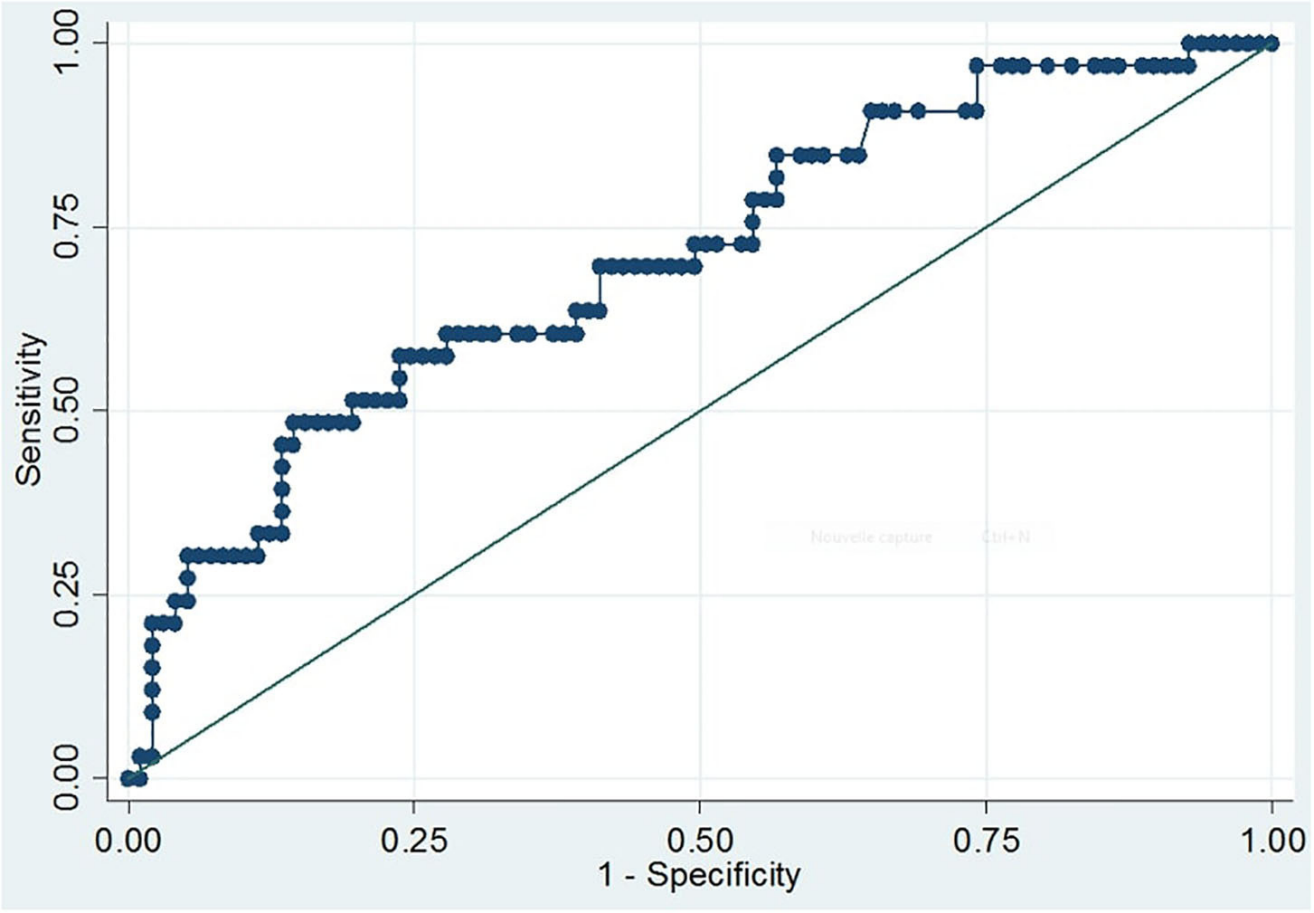
ROC curve of S100B serum levels for prediction of intracranial injury revealed by CT scans. The curve depicts the sensitivity and one-specificity values calculated for each individual serum S100B concentration with respect to the radiological findings of the initial CT scan. The quality of the discriminative potential is expressed by the AUC, which was 0.71 (95% CI, 0.60–0.81; *p* < 0.001).

The area under the curve (AUC) was calculated to evaluate the ability of S100B serum concentrations to diagnose CT+ patients. For all patients, AUC was 0.71 (95% CI; 0.60–0.81; *p* < 0.001; Figure 1). At the internationally established cutoff of 0.1 μg/L (16, 18, 28), sensitivity was 97% (95% CI; 84.2–99.9), specificity was 11% (95% CI; 5.8–19.4), the positive predictive value was 27% (95% CI; 19.3–36.1), and the negative predictive value was 92% (Table 2). The best threshold to achieve sensitivity of 100% (95% CI; 89.4–100) was 0.08 μg/L, with 7% specificity (95% CI; 2.3–13). The best threshold to achieve specificity of 31% (95% CI; 21.9–41.1) was 0.14 μg/L, with 91% (95% CI; 76–98) sensitivity.

**Table 2:** Contingency table of S100B serum (0.1 μg/L threshold) concentration according to cranial tomography findings.

**S100B**	**CT+**	**CT–**	
> 0.1 μg/L	32	87	Positive predictive value 27%, (19.3–36.1)
≤ 0.1 μg/L	1	10	Negative predictive value 92%, (61.5–99.8)
Total	33	97	130
	Sensitivity, 97% (84.2–99.9)	Specificity, 11% (5.8–19.4)	

*CT–, normal CT scan for mTBI patients with no signs of trauma-relevant intracranial lesions; CT+, abnormal CT scan for mTBI patients with at least one pathophysiological trauma-relevant intracranial lesion*.

Among the group of skiers (87 patients), AUC was 0.70 (95% CI; 0.56–0.84; *p* < 0.001). At a cutoff of 0.1 μg/L, sensitivity and specificity were 95% (95% CI; 75.1–99.9) and 10% (95% CI; 4.3–20.3), respectively. Positive predictive value was 24.1% (95% CI; 15.1–35.0) and negative predictive value was 87.5% (95% CI; 47.3–99.7). The best threshold for achieving sensitivity of 100% (95% CI; 83.2–100) was 0.08 μg/L, with 6% (95% CI; 2.0–15.0) specificity. The best threshold for achieving specificity of 33% (95% CI; 22.0–45.0) was 0.14 μg/L, with 85% (95% CI; 62.0–97.0) sensitivity.

### Clinical Factors' Influence on Serum S100B Levels

The median concentrations of serum S100B did not differ according to sex, age, and GCS score (13 vs. 14 and 15) (Table 3).

**Table 3:** Median concentrations of S100B according to demographic characteristics, clinical evaluation, radiological findings, and mechanism of injury.

		**n**	**S100B median (μg/L) (min; max; IQR)**	***p***	***P2***
**Demographics**					
Male		49	0.19 (0.05; 1.32; 0.14–0.37)	NS	NS
Female		81	0.22 (0.07; 1.42; 0.14–0.34)		
<65 years old		101	0.21 (0.05; 1.42; 0.14–0.35)	NS	NS
≥ 65 years old		29	0.21 (0.08; 1.00; 0.15–0.34)		
**Clinical and radiological evaluation**					
GCS score of 13–14		22	0.26 (0.05; 1.42; 0.11–0.38)	NS	NS
GCS score of 15		108	0.20 (0.07; 1.32; 0.14–0.35)		
CT–		97	0.18 (0.05; 1.42; 0.13–0.29)	<0.001	NA
CT+		33	0.35 (0.08; 1.32; 0.17–0.53)		
No fractures (F0)		96	0.20 (0.05; 1.42; 0.13–0.34)	F1F2 vs. F0: 0.036	F1F2 vs. F0: NS
	F1F2	34	0.27 (0.08; 1.32; 0.16–0.44)	F1 vs. F0: NS	F1 vs. F0: NS
	F1	26	0.23 (0.09; 0.85; 0.16–0.85)	F2 vs. F0: 0.041	F2 vs. F0: 0.009
Fractures	F2	8	0.96 (0.08; 1.32; 0.31–1.2)	F2 vs. F1: 0.048	F2 vs. F1: 0.009
Non-polytrauma		118	0.20 (0.05; 1.42; 0.14–0.35)	0.004	NS
Polytrauma		12	0.52 (0.08; 1.32; 0.27–1.10)		
**Injury mechanism**					
Non-skiers		43	0.27 (0.05; 1.42; 0.14–0.41)	0.19	NA
Skiers		87	0.19 (0.05; 1.21; 0.13–0.34)		
Using a helmet		76	0.17 (0.07; 1.21; 0.10–0.52)	NS	NA
Not using a helmet		11	0.20 (0.05; 1.2; 0.14–0.30)		

*A p-value of ≤ 0.05 is considered statistically significant. The multivariable analysis did not include non-significant results of the univariate analysis and did not include CT+. p, p-value of the median comparison; p2, p-value of the multivariate model; NA, Non Applicable; NS, Non Significant; GCS, Glasgow Coma Scale; CT–, negative computed tomography scan; CT+, positive computed tomography scan; F0, patients without fractures; F1, only one fracture; F2, two fractures or more; F1F2, F1 and F2 patients*.

A statistically significant difference was observed when comparing the median concentrations of patients with a negative CT scan (CT–) and that of abnormal CT scan (CT+) patients (*p* < 0.001). S100B medians were 0.18 μg/L (min: 0.05; max: 1.21; IQR: 0.13–0.28) and 0.31 μg/L (min: 0.08; max: 1.2; IQR: 0.17–0.62), respectively (Table 3).

To investigate whether peripheral traumatic injuries affect serum S100B concentrations, patients were sorted into groups depending on the number of fractures they suffered and whether they were categorized as polytrauma patients. The median S100B concentration was 0.20 μg/L (min: 0.05; max: 1.42; IQR: 0.13–0.34) for patients without fractures and 0.27 μg/L (min: 0.08; max: 1.32; IQR: 0.16–0.44) for patients that suffered at least one fracture. There was a statistically significant difference between the two values (*p* = 0.036). The medians for non-polytrauma patients and polytrauma patients were 0.20 μg/L (min: 0.05; max: 1.42; IQR: 0.14–0.35) and 0.52 μg/L (min: 0.08; max: 1.10; IQR: 0.27–1.10), respectively. Again, the difference was statistically significant (*p* = 0.004; Table 3).

The medians for patients with ski-related accidents and non-skiers were compared to determine whether ski practice has an effect on S100B measurements. There was no significant difference between the two subgroups. The use of a helmet did not impact S100B medians (Table 3).

A multivariate linear model was used to investigate the influence of different variables, such as age, sex, GCS score, number of fractures, and polytrauma, on S100B levels (see *p*2 in Table 3). The model revealed that age, sex, and GCS score did not have an impact on S100B levels. Among the variables that showed a difference when the S100B medians were compared (i.e., fractures and polytrauma), the only statistically significant association was observed in cases with two or more fractures (*p* = 0.009; Table 3).

**Figure 2:**
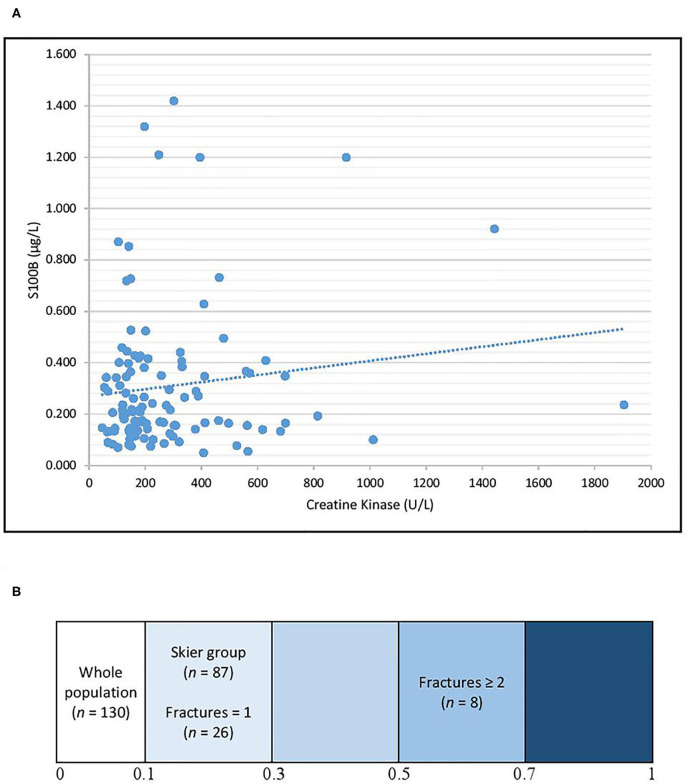
Correlation between S100B and CK for the whole study population **(A)** and heatmap representing the Spearman's correlation coefficients for different subgroups **(B)**. The Spearman's correlations coefficients were 0.07 for the whole population, 0.19 for the skier group, 0.13 for patients that suffered only one fracture, and 0.57 for patients that suffered at least two fractures.

To investigate the possible peripheral release of S100B due to tissue damage other than head trauma, including muscle damage, serum CK was measured. The Spearman's correlation coefficients between serum CK and serum S100B levels were 0.07 (*p* = 0.48) for the whole population, 0.19 (*p* = 0.11) for the skier group, 0.13 (*p* = 0.50) for patients that suffered only one fracture, and 0.57 (*p* = 0.19) for patients that suffered at least two fractures (Figure 2).

## Discussion

This prospective pilot study was the first to analyze the utility of S100B measurement for early management of mTBI patients in emergency departments in close proximity to ski stations. S100B is a well-established blood biomarker for mTBI screening in patients admitted in the ED. It is considered useful due to its negative predictive value and ability to achieve an approximate 30% reduction in unnecessary CT scans for these patients. In the present study, the diagnostic performance of serum S100B, which was always identified as significant by ROC curves, showed that, at a threshold of 0.1 μg/L, the sensitivity was 97% due to one patient from the skier group who had a negative S100B value and a positive CT scan showing a unique minimal hemorrhagic focus, which did not require neurosurgical care and did not have an impact on the clinical outcome, the patient was discharged after being held for observation. For this patient, the time interval for S100B sampling was >3 h and the value was confirmed by a second measurement of the same sample. We also found that, at a threshold of 0.1 μg/L, serum S100B levels would reduce unnecessary CT scans by only 10%. Allouchery et al. (5) observed a 30% scans reduction using French recommendation for CT Scan (29), while Minkkinen et al. (30) reported a 27.4% CT scans reduction when using S100B in adherence to the Scandinavian guidelines for mTBI with low risk of lesions. Theoretically, including subgaleal hematomas which do not constitute an intracranial injury would have reduced false positives in this study. The increased number of false-positive patients in this population could be associated with selection bias due to the hospital's geographic location and the nature of the accidents leading to ED admission. In Allouchery et al.'s (5) study, 862 (59.5%) patients of the whole population (*n* = 1,449) were admitted to the ED due to mTBI after a domestic fall. In contrast, in the present study, only 14 (10.8%) of the 130 patients in our study population suffered from domestic falls. We hypothesize that the lack of specificity of S100B for positive CT scans in the present study is linked to the underrepresentation of domestic accidents in comparison to studies conducted in urban areas, which lead to less complicated mTBIs and less increased S100B levels. The inclusion of these mTBI patients may vary depending on the hospital's decision algorithm to perform CT scans. The Canadian CT Head Rule may explain a lower specificity. In the present study's population, most mTBI admissions were related to ski accidents (66.9% of our population) rather than domestic falls. The ROC curve analysis showed a 2-fold specificity reduction in the skier group compared with the specificity for the whole population. This suggests that ski practice contributes to the increase in serum S100B, thus increasing false-positive S100B measurements when screening for mTBIs.

This hypothesis has been verified in various other sports (21), but not in ski practice. However, comparison of the median S100B concentrations between the skier and non-skier groups did not confirm this hypothesis. However, we cannot exclude this hypothesis based solely on median comparison, the non-skier group was not a valid control group as the patients in the two groups did not suffer the same injuries, ski practice is not the only variable that differs between the two groups, and the number of patients included in the skier and non-skier groups (87 and 43) do not provide sufficient statistical power. A valid way to investigate the relation of S100B to ski practice would be to measure S100B in a control group before and after ski practice.

Finally, previous studies have found that serum S100B concentrations were higher in the elderly population (31) (over 65 years old) and a threshold of 0.1 μg/L allowed for more false positives (26). However, this was not confirmed by our results. The poorer specificity of S100B observed in older patients by Allouchery et al. (5) was observed in all patients in the present study. This could also could be explained by the contribution of S100B from adipose tissue due to lipolysis or chondrocyte membrane rupture during ski practice (21).

Serum S100B in this present population is clearly affected by the presence of multiple fractures and polytrauma. Numerous studies suggest an extracerebral release of S100B in acute bone fractures patients with or without cerebral injuries (32–34). Though our multivariate model shows a stronger link between S100B and fractures than between S100B and polytrauma. The factor that most influenced S100B level was the presence of at least two fractures, as observed in the multivariate analysis. The hypothesis of an extracerebral S100B release is supported by the statistically significant difference in median serum S100B concentrations when comparing the no fracture and fracture subgroups and the improvement of the correlation factor between CK and S100B for patients with more fractures. Our results suggest that S100B should not be used as a head trauma biomarker when patients suffer from bone fractures. Although serum CK is not a valid biomarker for bone fracture exploration, we retained it as a practical and accessible biomarker to approximate peripheral traumatic lesions. In addition, we expected lower S100B levels in skiers using a helmet. However, the use of a helmet did not yield lower levels of S100B in this study. This can be explained by the S100B release consequent to extracranial injuries counterbalancing the potential protective effects of the helmet. Similar results were described for bicycle-related trauma (35). Another hypothesis is that in skiers, mTBI is consecutive to a combination of direct head impact, which should be protected by the helmet, and blunt head injury via acceleration-deceleration phenomena which would not be affected by the use of a helmet. Neural tissue is susceptible to injury from shearing stresses which are less tolerated than uniform compressive and tensile forces (36).

The investigation of other potential mTBI biomarkers, such as glial fibrillary acidic protein (GFAP), heart fatty acid binding protein (H-FABP), and ubiquitin carboxy-terminal hydrolase L1 (UCH-L1), may enable the development of more neurospecific tools, bringing new perspectives for mTBI management. In Yue et al.'s (37) study of suspected TBI patients, blood GFAP concentrations allowed the prediction of pathological findings in MRI in patients with a negative CT scan. In Lagerstedt et al.'s (38) study of mTBI patients with a GCS score of 15, a panel combining H-FABP, GFAP, and interleukin 10 (IL-10) yielded a 52% specificity (38). Posti et al. (39) tested a panel of three biomarkers—S100B, tau, and H-FABP—with 100% sensitivity and 46.4% specificity. Thus, use of a biomarker panel may have the potential to reduce unnecessary CT scans, and could be particularly interesting in mTBI consecutive to a complicated mechanism and/or when fractures are present. The S100B protein is to this day the only brain trauma biomarker used in routine in Europe and this study was conducted to explain the limited performances of S100B in emergency departments where ski-related accidents represent a significant percentage. Although, testing other biomarkers would have been interesting, test kits are not yet available in routine kits with the proper CE marking.

## Limitations

The main limitation of our study is the relatively small number of patients included. However, S100B is a robust biomarker and the ~30% CT scan reduction was confirmed by numerous studies regardless of the population size (5, 16, 18, 19). Also, a multicenter design would have provided a preferable basis for subsequent generalization of our findings. Regarding the data collection and interpretation, traceability concerning patients' inclusion process has not been documented, this constitutes a potential selection bias. Additionally, there was a lack of documentation of some clinical variables justifying the use of a CT scan such as the duration of loss of consciousness and posttraumatic amnesia. Finally, the inclusion of subgaleal hematomas in the abnormal CT scans may influence the comparability to other studies which don't include this CT finding, although, we did not observe a statistical difference in the interpretation of our data when including or excluding subgaleal hematomas from the positive CT scans.

## Conclusion

S100B is a well-established blood biomarker for early mTBI management. When used for clinical evaluation, it enables a significant reduction in the number of CT scans performed. Our study shows that the utility of S100B could vary depending on multiple confounding factors, such as sport practice and bone fractures. For ski-related mTBIs, S100B does not allow a significant reduction of unnecessary CT scans.

## Data Availability Statement

The datasets generated for this study are available on request to the corresponding author.

## Ethics Statement

The studies involving human participants were reviewed and approved by CER-VD. The patients/participants provided their written informed consent to participate in this study.

## Author Contributions

SK and PS analyzed and interpreted the data and wrote the initial version of the manuscript. DB and VS designed the study and assisted with interpretation of the data and writing of the manuscript. LP, JC, and VF supervised the trial and data collection. CO, JD, and LA carried out assays. BP provided statistical advice for the study design and analyzed the data. All authors contributed to the article and approved the submitted version.

## Conflict of Interest

The authors declare that the research was conducted in the absence of any commercial or financial relationships that could be construed as a potential conflict of interest.

## References

[1] Carroll L Cassidy JD Holm L Kraus J Coronado V Methodological issues and research recommendations for mild traumatic brain injury: the who collaborating centre task force on mild traumatic brain injury. J Rehabil Med. (2004) 36:113–25. 10.1080/16501960410023877 15083875

[2] Cassidy JD Carroll L Peloso P Borg J von Holst H Holm L Incidence, risk factors and prevention of mild traumatic brain injury: results of the who collaborating centre task force on mild traumatic brain injury. J Rehabil Med. (2004) 36:28–60. 10.1080/16501960410023732 15083870

[3] Tagliaferri F Compagnone C Korsic M Servadei F Kraus J. A systematic review of brain injury epidemiology in Europe. Acta Neurochir. (2006) 148:255–68. 10.1007/s00701-005-0651-y 16311842

[4] Peeters W van den Brande R Polinder S Brazinova A Steyerberg EW Lingsma HF. Epidemiology of traumatic brain injury in Europe. Acta Neurochir. (2015) 157:1683–96. 10.1007/s00701-015-2512-7 26269030 PMC4569652

[5] Allouchery G Moustafa F Roubin J Pereira B Schmidt J Raconnat J. Clinical validation of S100B in the management of a mild traumatic brain injury: issues from an interventional cohort of 1449 adult patients. Clin Chem Lab Med. (2018) 56:1897–904. 10.1515/cclm-2018-0471 29924734

[6] Statistique, 2018 des situations d'urgence en montagne (n,.d.). Retrieved from: https://www.sac-cas.ch/fileadmin/Ausbildung_und_Wissen/News/2018/Bergnotf%C3%A4lle_Schweiz_2018_Internet_FR.pdf (accessed February 14, 2020).

[7] Mutch CA Talbott JF Gean A. Imaging evaluation of acute traumatic brain injury. Neurosurg Clin N Am. (2016) 27:409–39. 10.1016/j.nec.2016.05.011 27637393 PMC5027071

[8] Vos PE Alekseenko Y Battistin L Ehler E Gerstenbrand F Muresanu DF. Mild traumatic brain injury: mild traumatic brain injury. Eur J Neurol. (2012) 19:191–8. 10.1111/j.1468-1331.2011.03581.x 22260187

[9] Pearce MS Salotti JA Little MP McHugh K Lee C Kim KP. Radiation exposure from CT scans in childhood and subsequent risk of leukaemia and brain tumours: a retrospective cohort study. Lancet. (2012) 380:499–505. 10.1016/S0140-6736(12)60815-0 22681860 PMC3418594

[10] Reinus WR Wippold FJ Erickson KK. Practical selection criteria for noncontrast cranial computed tomography in patients with head trauma. Ann Emerg Med. (1993) 22:1148–55. 10.1016/S0196-0644(05)80981-3 8517566

[11] Nagy KK Joseph KT Krosner SM Roberts RR Leslie CL Dufty K. The utility of head computed tomography after minimal head injury. J Trauma. (1999) 46:268–70. 10.1097/00005373-199902000-00012 10029032

[12] Haydel MJ Preston CA Mills TJ Luber S Blaudeau E DeBlieux PM. Indications for computed tomography in patients with minor head injury. New Engl J Med. (2000) 343:100–5. 10.1056/NEJM200007133430204 10891517

[13] Miller EC Holmes JF Derlet RW. Utilizing clinical factors to reduce head CT scan ordering for minor head trauma patients. J Emerg Med. (1997) 15:453–7. 10.1016/S0736-4679(97)00071-1 9279694

[14] Stein SC Ross SE. The value of computed tomographic scans in patients with low-risk head injuries. Neurosurgery. (1990) 26:638–40. 10.1227/00006123-199004000-00012 2330085

[15] Oris C Pereira B Durif J Simon-Pimmel J Castellani C Manzano S. The biomarker S100B and mild traumatic brain injury: a meta-analysis. Pediatrics. (2018) 141:e20180037. 10.1542/peds.2018-0037 29716980

[16] Undén J Romner B. Can low serum levels of S100B predict normal CT findings after minor head injury in adults?: An evidence-based review and meta-analysis. J Head Trauma Rehabil. (2010) 25:228–40. 10.1097/HTR.0b013e3181e57e22 20611042

[17] Undén J Romner B. Scandinavian guidelines for initial management of minimal, mild and moderate head injuries in adults: an evidence and consensus-based update. BMC Med. (2013) 11. 10.1186/1741-7015-11-50 23432764 PMC3621842

[18] Biberthaler P Linsenmeier U Pfeifer K-J Kroetz M Mussack T Kanz K-G. Serum S-100B concentration provides additional information fot the indication of computed tomography in patients after minor head injury: a prospective multicenter study. Shock. (2006) 25:446–53. 10.1097/01.shk.0000209534.61058.35 16680008

[19] Bouvier D Oddoze C Ben Haim D Moustafa F Legrand A Alazia M. Intérêt du dosage sérique de la protéine S100B dans la prise en charge du patient après traumatisme crânien léger. Ann Biol Clin. (2009) 67:425–31. 10.1684/abc.2009.0347 19654082

[20] Calcagnile O Undén L Undén J. Clinical validation of S100B use in management of mild head injury. BMC Emerg Med. (2012) 12:13. 10.1186/1471-227X-12-13 23102492 PMC3527238

[21] Schulte S Podlog LW Hamson-Utley JJ Strathmann FG Strüder HK. A systematic review of the biomarker S100B: implications for sport-related concussion management. J Athlet Train. (2014) 49:830–50. 10.4085/1062-6050-49.3.33 25299445 PMC4264656

[22] Bieniek KF Ross OA Cormier KA Walton RL Soto-Ortolaza A Johnston AE. Chronic traumatic encephalopathy pathology in a neurodegenerative disorders brain bank. Acta Neuropathol. (2015) 130:877–89. 10.1007/s00401-015-1502-4 26518018 PMC4655127

[23] McKee AC Stein TD Nowinski CJ Stern RA Daneshvar DH Alvarez VE. The spectrum of disease in chronic traumatic encephalopathy. Brain. (2013) 136:43–64. 10.1093/brain/aws307 23208308 PMC3624697

[24] Stern RA Riley DO Daneshvar DH Nowinski CJ Cantu RC McKee AC. Long-term consequences of repetitive brain trauma: chronic traumatic encephalopathy. PM&R. (2011) 3:S460–7. 10.1016/j.pmrj.2011.08.008 22035690

[25] Stiell IG Wells GA Vandemheen K Clement C Lesiuk H Laupacis A. The Canadian CT Head Rule for patients with minor head injury. Lancet. (2001) 357:1391–6. 10.1016/S0140-6736(00)04561-X 11356436

[26] Laribi S Kansao J Borderie D Collet C Deschamps P Ababsa R. S100B blood level measurement to exclude cerebral lesions after minor head injury: the multicenter STIC-S100 French study. Clin Chem Lab Med. (2014) 52:527–36. 10.1515/cclm-2013-0621 24225131

[27] Lew HL Otis JD Tun C Kerns RD Clark ME Cifu DX. Prevalence of chronic pain, posttraumatic stress disorder, and persistent postconcussive symptoms in OIF/OEF veterans: polytrauma clinical triad. J Rehab Res Dev. (2009) 46:697–702. 10.1682/JRRD.2009.01.0006 20104399

[28] Zongo D Ribéreau-Gayon R Masson F Laborey M Contrand B Salmi LR. S100-B protein as a screening tool for the early assessment of minor head injury. Ann Emerg Med. (2012) 59:209–18. 10.1016/j.annemergmed.2011.07.027 21944878

[29] Jehlé E Honnart D Grasleguen C Bouget J Dejoux C Lestavel P Traumatisme crânien léger (score de Glasgow de 13 à 15): triage, évaluation, examens complémentaires et prise en charge précoce chez le nouveau-né, l'enfant et l'adulte. Annal Franç Méd d'Urgence. (2012) 2:199–214. 10.1007/s13341-012-0202-4

[30] Minkkinen M Iverson GL Kotilainen A-K Pauniaho S-L Mattila VM Lehtimäki T. Prospective validation of the scandinavian guidelines for initial management of minimal, mild, and moderate head injuries in adults. J Neurotrauma. (2019) 36:2904–12. 10.1089/neu.2018.6351 31111795

[31] Calcagnile O Holmén A Chew M Undén J. S100B levels are affected by older age but not by alcohol intoxication following mild traumatic brain injury. Scand J Trauma Resuscit Emerg Med. (2013) 21:52. 10.1186/1757-7241-21-52 23830006 PMC3704936

[32] Anderson RE Hansson LO Nilsson O Dijlai-Merzoug R Settergren G. High serum S100B levels for trauma patients without head injuries. Neurosurgery. (2001) 48:1255–8; discussion 1258-1260. 10.1227/00006123-200106000-00012 11383727

[33] Savola O Pyhtinen J Leino TK Siitonen S Niemelä O Hillbom M. Effects of head and extracranial injuries on serum protein S100B levels in trauma patients. J Trauma. (2004) 56:1229–34; discussion 1234. 10.1097/01.TA.0000096644.08735.72 15211130

[34] Undén J Bellner J Eneroth M Alling C Ingebrigtsen T Romner B. Raised serum S100B levels after acute bone fractures without cerebral injury. J Trauma. (2005) 58:59–61. 10.1097/01.TA.0000130613.35877.75 15674151

[35] Thelin EP Zibung E Riddez L Nordenvall C. Assessing bicycle-related trauma using the biomarker S100B reveals a correlation with total injury severity. Eur J Trauma Emerg Surg. (2016) 42:617–25. 10.1007/s00068-015-0583-z 26490563

[36] Cantu RC Head injuries in sport. Br J Sports Med. (1996) 30:289–96. 10.1136/bjsm.30.4.289 9015588 PMC1332409

[37] YueJKYuhELKorleyFKWinklerEASunXPufferRC. Association between plasma GFAP concentrations and MRI abnormalities in patients with CT-negative traumatic brain injury in the TRACK-TBI cohort: a prospective multicentre study. Lancet Neurol. (2019) 18:953–61. 10.1016/S1474-4422(19)30282-031451409

[38] Lagerstedt L Egea-Guerrero JJ Bustamante A Rodríguez-Rodríguez A El Rahal A Quintana-Diaz M. Combining H-FABP and GFAP increases the capacity to differentiate between CT-positive and CT-negative patients with mild traumatic brain injury. PLoS ONE. (2018) 13:e0200394. 10.1371/journal.pone.0200394 29985933 PMC6037378

[39] Posti JP Takala RSK Lagerstedt L Dickens AM Hossain I Mohammadian M. Correlation of blood biomarkers and biomarker panels with traumatic findings on computed tomography after traumatic brain injury. J Neurotrauma. (2019) 36:2178–89. 10.1089/neu.2018.6254 30760178 PMC6909751

